# Continuous blood glucose monitoring prediction for diabetes using evolving neural network

**DOI:** 10.1038/s41598-025-29169-x

**Published:** 2025-11-25

**Authors:** Neil Vaughan

**Affiliations:** 1 Department of Clinical and Biomedical Sciences (CBS), University of Exeter, Exeter, UK; 2 Exeter Centre of Excellence for Diabetes Research (ExCEeD), Exeter, UK; 3 Royal Academy of Engineering (RAEng), Research Fellowship, London, UK

**Keywords:** Diabetes, Blood glucose, Forecast, CGM, Predictive medicine, Endocrine system and metabolic diseases, Computational science

## Abstract

This research presents a new evolving neural network approach to forecast blood glucose for people with diabetes. The accuracy of forecasting using the proposed evolving neural network is demonstrated to outperform a conventional back propagation neural network. People with diabetes need to control their blood sugar levels. High blood sugar over long term leads to many other health complications. To avoid high blood sugar, it is important for people to be able to predict what will happen to blood sugar so that they can do something to prevent hypo or hyper glycaemia. However, many external uncontrollable factors can make blood glucose difficult to predict, such as meals which increase carbs and glucose goes up. Exercise also affects blood glucose, but exercise can be aerobic or anaerobic and these affect blood glucose in opposite ways. There has been research aiming to predict blood glucose by analysing previous recorded data from continuous glucose monitoring devices. This research applies a new approach with evolutionary computation to evolve a neural network, using neuro evolution, and the optimised neural network is then applied to predict and forecast blood glucose changes. In the comparison of accuracy, the results show that evolved neural network outperformed a back-propagation neural network in this task on forecasting CGM data. This can help people with diabetes to have a better idea about how their blood glucose is going to change before it occurs, so that hypo and hyper can be avoided. This can reduce diabetes complications and costs for the health service.

## Introduction

### Type 1 and type 2 diabetes

Diabetes mellitus (DM) is a condition that affects over eight hundred million people globally. Diabetes is known as the fastest growing public health concern [Hossain et al.^1^. This condition is characterised by hyperglycaemia resulting from a defect in insulin secretion or action. Insulin normally facilitates the entrance of glucose into cells for energy use, but when this process is impaired, glucose accumulates in the blood. DM poses a global health challenge. Diabetes affected over 380 million people worldwide by 2014, according to the International Diabetes Federation (IDF), which has increased to over 800 million in 2025 according to the World Health Organisation (WHO) [Pan et al.^2^. There are two main types of DM: Type 1 (T1DM) and Type 2 (T2DM). T1DM is an autoimmune disorder that destroys insulin-producing cells and often manifests in childhood or adolescence. T2DM, the most prevalent form, typically develops in adults and is associated with insulin resistance. All types of DM require blood glucose control to mitigate short-term and long-term complications, including blindness, kidney disease, and neuropathy.

### Difficulties with maintaining and predicting blood glucose

Maintaining effective management of diabetes and blood sugar is crucial to prevent not only severe long-term complications but also hazardous short-term situations. Individuals with diabetes must determine the correct insulin dosage, thus requiring the ability to estimate their glucose levels after a meal. Patients with T1DM must calculate insulin doses to manage carbohydrate intake and prevent glucose spikes. Many rely on approximations or generic predictors, along with capillary blood glucose measurements before meals.

Estimating and predicting blood glucose changes are hard for several reasons, such as many interacting factors affecting glucose simultaneously including meals, carbs, aerobic and anaerobic exercise. Additional factors like stress or sleep cycle also influence glucose levels, making control challenging. Controlling glycemia can be particularly challenging in certain populations, such as growing adolescents, individuals with comorbidities, and pregnant women. In such cases, personalised models predicting glucose levels based on individual factors could improve management.

Research indicates significant variability in individual responses to identical meals, highlighting gaps in understanding the relationship between meals, insulin administration, and blood glucose values. Addressing this complexity requires the development of formal models to predict future blood glucose concentrations based on current glucose levels, carbohydrate intake, and insulin administration.

### Finger prick data for blood glucose predictions

The data that patients use to monitor and predict their glucose conventionally comes from finger prick, or capillary blood glucose measurements, which can be taken manually at certain times such as before meals, before sleep or near exercise times. However, these are often only taken once or twice a day, which does not give any indication of what happens to blood glucose throughout the rest of the day and night when BG is not being monitored.

### Continuous glucose monitoring data for BG prediction

Recently, to improve on the frequency of blood glucose data collection, continuous glucose monitoring (CGM) systems were introduced. These CGM devices can collect data continuously throughout the day and night. There are various common manufacturers including Dexcom and Libre. The CGM data can commonly be collected at 5–15 min intervals, which varies between devices and may affect glucose control metrics [Russon et al.^3,4^. The CGM data could improve the ability to predict BG response to certain events but as the interval between measurements becomes smaller, the amount of data to process increases and with a large number of patients, it can be a burden on clinicians or health services to provide continuous data analysis for all patients. Continuous glucose monitoring (CGM) systems and insulin pumps have also been connected together as a closed-loop system, to automate insulin delivery at the required levels, which has shown promise to assist in glucose management, may require less input from the patient or clinician, but still requires careful monitoring and analysis of data.

To contextualise this project within the broader literature of blood glucose prediction, there have been many different types of algorithms to perform blood glucose prediction. This includes different prediction horizons including 30 min, 1 h, 2 h, 4 h or more. There have also been a range of different applications, such as relating to closed-loop systems, prediction around meal times when glucose varies, prediction around exercise times and other applications. Additionally, various prediction methods have been proposed, from simple time-series models like AR, ARX, ARMAX, to other more complex prediction frameworks.

Machine learning techniques and frameworks have recently been employed in various research to predict blood sugar levels in diabetic patients. Recent approaches of predicting blood glucose have utilised data gathered from real patients continuously through a CGM device. One particular type of machine learning which has shown outstanding promise for BG prediction using CGM data is evolutionary algorithms and genetic programming.

### Aims of this paper

This paper summarises the current state of art in evolutionary computation for blood glucose prediction. Furthermore we propose a new evolutionary algorithm approach in which evolutionary computation is employed for the optimisation of neural networks. Once training is completed, the evolved neural networks are subsequently applied to predict and forecast blood sugar. The accuracy results from the evolved neural network are then compared to other leading methods of BG prediction.

## Review of evolutionary computation BG forecasts

### Search terms on evolutionary computation BG prediction

To thoroughly identify all related research which has applied evolutionary computation for BG Prediction, a literature search was conducted using the PubMed and IEEE Xplore databases. The keyword searches with various terms included (“evolutionary computation” OR “genetic algorithms,” OR “genetic programming,”) AND (“diabetes” OR “glucose control” OR “glucose monitoring”.)

### Related work on evolutionary computation BG prediction

Neuro-evolution is a great contender as an alternative to the deep learning methods, which shows promise to be successfully applied for time series forecasting models. Research by De Falco et al.^5^ has aimed to achieve prediction of personalised blood glucose levels in Type 1 diabetes (T1D) patients using a neuro-evolution approach. They exploit a neuro-evolutionary algorithm to model and predict future personalised blood glucose levels which were comparable to other state-of-the-art methods.

Other areas of health AI research recently perform well with healthcare data including using transformer models for Alzheimer’s disease diagnosis [Lu et al.^6^, tuberculosis classification [Lu et al.^7^, COVID-19 classification [Zhu et al.^8^, or deep learning networks for classifying blood cells [Zhu et al.^9^. A main focus on the machine learning frameworks for health applications is around explainability [Chadaga et al.^10–12^. The purpose of this is that predictions of blood glucose levels or any other health metrics need to be shown why the predictions have been made in a human understandable method that clinicians would be able to agree with.

Particularly for multi-objective decision making, neuro-evolution-based computing paradigms are currently performing well previously with various datasets and modern scenarios, including automation of healthcare or industrial processes [Wang et al., 2025], health data science studies including the COVID-19 transposition [Shoaib et al.^13^, and neuro-evolution also shows promise for automating Intelligent home or point-of-care household health robots [Sun et al.^14^.

Evolutionary computation has been used to forecast CGM data for diabetes. Colmenar et al.^15^ collected input data from continuous glucose monitoring system, the estimated carbohydrate count, and insulin administration data, which was fed into a genetic programming model which considered prediction of blood glucose forecast with horizons of 30, 60, 90 and 120 min.

Hamdi et al.^16^ aimed to achieve accurate prediction of continuous blood glucose based on differential evolution algorithm. This approach does not require the patient to specific their daily activities: meal intake, insulin injection and emotional factors, which can be error prone. Instead the method uses only continuous glucose monitoring (CGM) data to predict blood glucose levels independently of other factors applying differential evolution (DE) algorithms. They obtained average of root mean square error (RMSE) 9.44 mg/dL for prediction horizon (PH) of 15 min and also achieved good results for PH of 30, 45 and 60 min.

Genetic BG forecast by Cervigón et al.^17^ used genetic algorithms to predict BG values with time horizon of 24 h. This method is implemented by dividing the day into 12 time intervals. Applying genetic algorithms in combination with ensemble approach provided robust predictions that take into account all the uncertainty associated with the interaction between insulin and glucose.

During recent years some attempts relying on evolutionary based algorithms have been advanced to derive diabetes modelling [Hidalgo et al.^18^, by using as input CGM values for extracting relationships under the form of explicit mathematical expressions, even on the basis of previous and estimated future carbohydrate intakes [Hidalgo et al.^19^.

Evolution has been proven as a suitable algorithm for predicting glucose levels [Velasco et al.^20,21^. One of the difficulties of applying differential evolution has been the difficulties in collecting enough training data and that each patient’s response is individual and can vary in a high degree due between people, due to a lot of personal factors which can be seen to influence the blood glucose in different ways. Evolution models have been developed model to predict glucose-after-meal behaviour. The evolutionary models based on different scenarios get more robust predictions, decreasing significantly the number of hazardous predictions. Also evolutionary algorithms performed well when training data was augmented by generative algorithm.

## Methods for evolutionary BG forecast system

### CGM data as a time series

Time series analysis is applied to datasets where the information is arranged sequentially based on the order of time. Continuous Glucose Monitoring (CGM) inherently generates time series data. As the data will be recorded continuously throughout the day and night, the CGM data will be reflecting glucose fluctuations, influenced by factors such as the time of day, meal intake, and physical activity. Time series analysis and forecasting has emerged as a valuable approach tailored for investigating CGM data. CGM time series analysis methods facilitate examination of CGM data as a sequential series over time.

In existing literature, numerous CGM data analysis tools and functions have been developed to effectively handle and process CGM data. These include time series functions designed to enhance missing data by applying interpolation [Russon et al.^3^ and identify outliers accurately to remove erroneous reading from sensor noise. Additionally, the available CGM tools calculate CGM metrics recommended by the international consensus for CGM [Danne et al.^22^ and essential time series parameters. Furthermore, some available CGM tools enable the creation of interactive graphs for direct visualisation of CGM data, either as a time series or in real-time, through integration with CGM device connectivity.

### Ethics regulations and consent

The CGM dataset used in this research originated from a secondary analysis of Type 1 Diabetes (T1D) participants who were recruited as part of the EXTOD education study [Narendran et al.^23^. The dataset is not currently publicly available. The EXTOD study was sponsored by Taunton and Somerset NHS Foundation Trust (reference 2511). The EXTOD study and all experimental protocols were approved by the institutional licensing committee and research ethics committee (REC) of West Midlands – Coventry and Warwickshire, (Reference: 16/WM/0034). The study was included within National Institute for Health Research (NIHR) Portfolio ID: 30478.

All experiments were performed in accordance with relevant guidelines and regulations. The trial was managed by the Leicester Clinical Trials Unit (LCTU) at the University of Leicester. This research was designed in accordance with the University of Exeter’s ethics committee and Internal Review Board (IRB) guidelines. This research was completed in accordance with the Declaration of Helsinki.

Informed consent was obtained from all subjects. One of the exclusion criteria was that if subjects had inability to give informed consent, they were excluded from the study. Prior to giving consent, participants were provided time to discuss the patient information with family, friends, their GP or the research nurse. Written consent was obtained by a suitably qualified research nurse designated by the chief investigator (CI).

### Dataset description

Participants were all adults aged 18–70 years. Participants were training for sports events including distance running. Participants were excluded if pregnant, using an insulin pump, had hypoglycemia unawareness, were unable to exercise, understand English or give informed consent. The remaining dataset consisted of 119 CGM traces from 72 participants. Participants wore Dexcom G6 CGM devices.

In pre-processing phase, the data has been anonymised to contain only the CGM values without identifying information. The post-processed data consists of two columns: the time stamp and the raw CGM value as mmol/L, saved within a .csv file which can be directly imported into Python. Participants wore the CGM device for approximately 2 weeks (13.8 days) which consists of about 3854 CGM values at 5-minute intervals. Several of the CGM files contained some missing values, where the device had not recorded consecutive reading. As a pre-processing step, missing values were computed using interpolation [Russon et al.^3^. Also the CGM data quite often (around 41% of values) were recorded with CGM interval of 5 min and 1 s interval, or 4 min 59 s interval, as well as exactly 5 min intervals. This slight interval variation was caused by an artifact of the CGM device itself and it did not affect the prediction task much as 1 s represents only 0.33% of 5 min which is a minor discrepancy.

### Description of problem in forecasting sequential BG

**Fig. 1:**
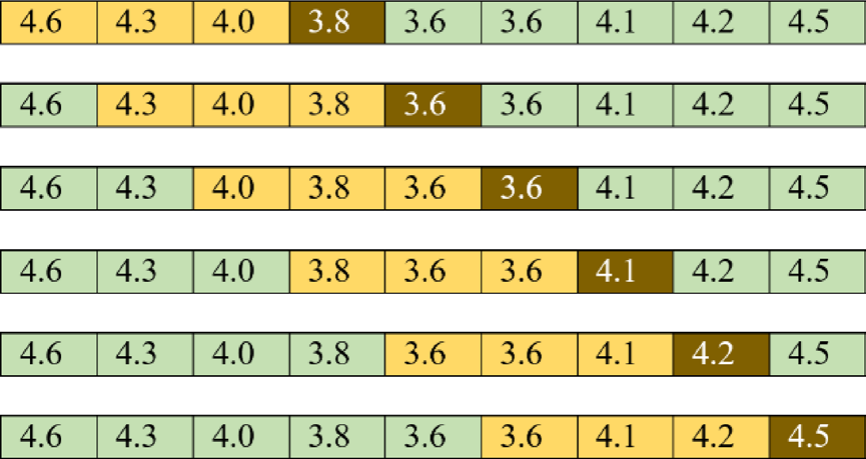
Sliding window within the CGM data used to predict the subsequent glucose value. The units of BG are in mmol/L.

In this research the forecasting of blood glucose was formulated into a problem definition as shown in Fig. 1 as described below. Initially, the model has been trained using fixed windows of sizes 3–10 and in future a window of adjustable size could be used. The window size is created from length 3 to 10 blood glucose values, shown as the yellow values in Fig. 1. In 5 min interval CGM data, a window of size 10 contains data recorded over the maximum of 45 min, whereas in 15 min interval data a window of size 10 would equate to 2 h and 15 min of CGM data. The window contains the preceding 3 to 10 values of blood glucose. The BG values within the window are used as the input data, to predict the subsequent unseen glucose value. The current prediction horizon of the model includes the next 1 step ahead, which is the next 5-minute reading. This can be useful in practice, for near-future forecasting and for example when manufacturer devices are generating the rate-of-change arrows, which allow users to see that the BG is currently likely to either be increasing or decreasing at the next timestep. We have in other publications already analysed data for prediction at longer term 30-60-120 min predictions using alternative ML methods [Russon et al.^24^. In Fig. 1, the future glucose value to be predicted is shown in brown. The values shaded green are not used until subsequent predictions.

### Structure of evolving artificial neural network (ANN)

For this forecasting task we used a feed-forward neural network. The feed-forward neural network is a type of artificial neural network (ANN). ANNs are inspired by the biological neural networks found in the brain. ANNs are designed to mimic the basic functioning of neurons, possess versatile capabilities including classification, regression, and various other techniques. Their widespread adoption in machine learning and artificial intelligence stems from their remarkable effectiveness across a diverse range of problem domains.

Each connection between neurons is represented by a weight, forming a matrix. Therefore, our input consists of an [*N*, *i*] matrix, where *N* denotes the number of observations to be processed, and *i* represents the number of variables, in the case of CGM, *i* = 1. During a forward pass through our network, we multiply the matrices together, apply the transition function at each layer, and obtain our result.

An addition and critical step of the forward pass process, involves adding a bias vector at each layer. This bias vector, denoted as an [1, *M*] vector, is incorporated into each observation following the activation function, where *M* represents the number of nodes for that particular layer.

Neural networks are typically trained using backpropagation, a process where the current error, such as Mean Squared Error (MSE) or Cross-Entropy, is propagated backward from the output layer through the hidden layers. This allows the weights to be adjusted iteratively to minimise the error. However, in our application, we will use a genetic algorithm instead of backpropagation to train the weights.

For the prediction of continuous glucose monitoring data, we are using a neural network to forecast time series data. In one-dimensional time series scenarios, there exists only a single variable *i* alongside a time index. While recurrent neural networks (RNNs) are commonly utilised to tackle time series problems, featuring a recurrent layer that feeds output back into the input layer for subsequent time index predictions, our project adopts an alternative approach. In this research, we use an alternative approach, by configuring the input layer as a ‘window’ (shown as the yellow values in Fig. 1). The window span a specific range of indices, which was restricted to length of between 3 and 10 glucose values, directly preceding the single future value to be predicted.

The example in Fig. 1 depicts a subset of the blood glucose time series prediction challenge. Our objective was to forecast the values in the brown boxes. To predict the value 3.8 in the first row, we input 4.6, 4.3 and 4.0 into our neural network’s input layer. In this example, our ‘window’ size is three, and so the three previous glucose values (shown in yellow) directly prior to the current value to be predicted (shown in brown) (non-inclusive) serve as input variables for prediction. The window size was varied during experimentation to identify the ideal size for the problem. For this task, we evaluated multiple different window sizes from 3 to 10. Our neural network also featured three hidden layers, each comprising 5 hidden nodes and utilising the ReLU activation function. However, the number of inputs and the number of input nodes are varied between 3–10 based on the ‘window’ size.

**Fig. 2:**
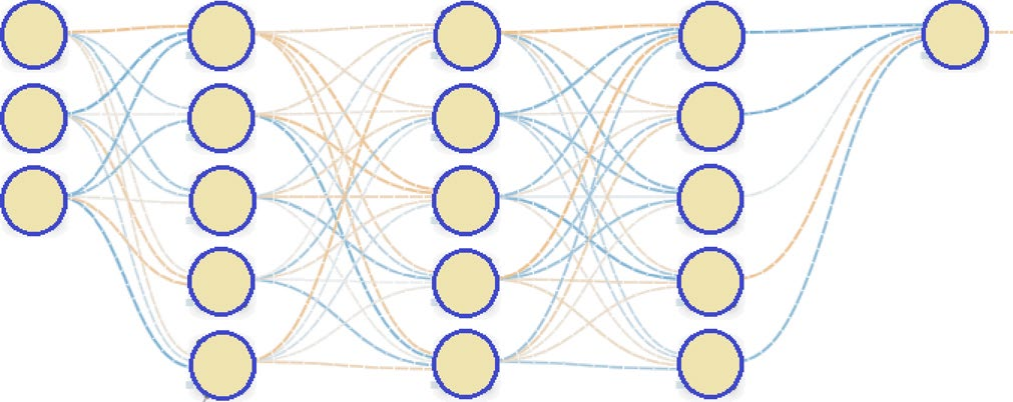
Structure (topology) of the neural network. In this situation there are 3 input nodes although this was varied, depending on the window size which is adjusted between 3 and 10. There are 3 hidden layers each with 5 hidden nodes. The one output node is used to predict the single future glucose value.

Due to the need to evolve the neural network weights, we developed the ANN code from scratch. Common libraries lacked the flexibility required for our purposes. Our implementation allows for experimentation with an arbitrary number of layers and nodes per layer. However, we restricted ourselves to using the ReLU activation function across all layers. We experimented with 3 input nodes, 3 hidden layers each with 5 nodes, and 1 output node as shown in Fig. 2).

### Description of over-fitting in CGM data forecasting

**Fig. 3:**
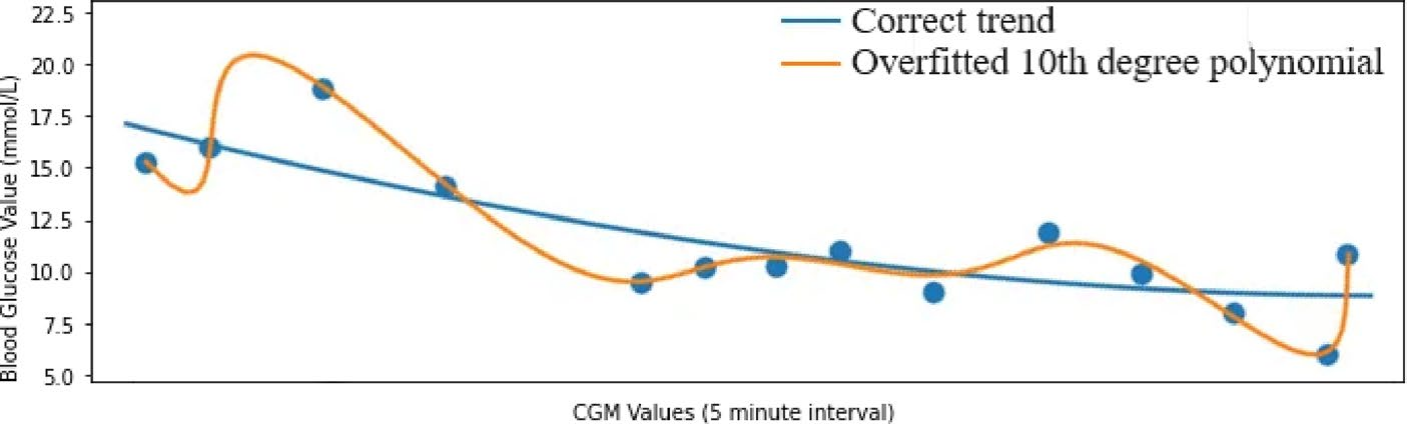
Example of overfitting. This CGM data example tends to have a gradual downward trend. However if a more complex polynomial model is used, the trend line can get closer to each individual datapoint, but it is only adapting to the noise in this particular training data and will perform badly on other unseen CGM datasets.

The proposed model implementation is also intended to avoid over fitting. The implementation aims to address one of the common pitfalls encountered in machine learning models, known as over-fitting. Over-fitting arises when models have an excessive number of adjustable parameters, leading them to essentially memorise the input data. Consequently, their performance deteriorates when predicting values they haven’t encountered before compared to the ones they were trained on. Figure 3 illustrates an instance of over-fitting, wherein the model artificially minimises the error of residuals by excessively increasing the degree of the polynomial. However, this approach results in hyper-parameterisation specific to the training data, failing to accurately capture the underlying trend. Hence, it produces highly erroneous predictions for unseen data.

### Method to prevent over-fitting

Our objective was to develop a model that is both simple and powerful, capable of accurately predicting new BG values. Various methods exist to prevent overfitting in neural networks, ranging from dropout layers to cross-validation. In this study, the method we employed uses early stopping measures to prevent overfitting. Overfitting is a general problem which occurs regularly in many domains, so we describe our approach in detail for reproducibility.

**Fig. 4:**
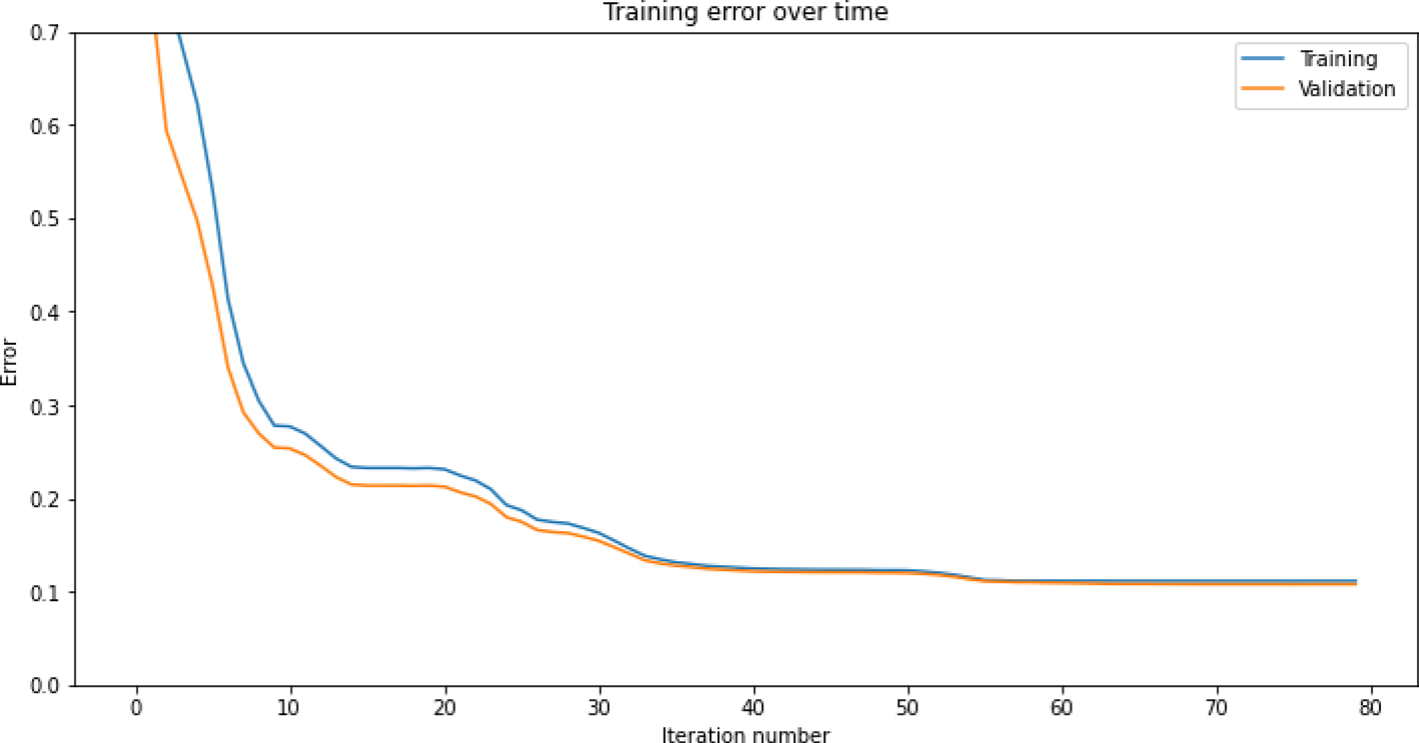
Graph plotting the the prediction error showing improvement over time durtring the evolution (training) of the neural network. When the validation accuracy starts to stagnate and gets worse, overfitting has occurred, so the process should be stopped early at this point.

To implement the early stopping, the dataset was first split into two main components: the training and testing datasets. The training dataset was used to train the model, while the testing dataset was used to assess its performance. As the model was trained, when overfitting began to occur—detected by an increase in error on the testing dataset—our algorithm was stopped early. The error over 80 iterations is shown in Fig. 4. This proactive approach prevents the model from excessively fitting to the training data, which may cause the error on the testing data to rise. Future work may also further explore alternative methods or training without early stopping.

Early stopping was triggered when the error on the testing dataset increased by a certain threshold after a predefined number of iterations, ensuring that overfitting is mitigated effectively. This strategy safeguards against the model’s tendency to continue improving performance on the training data while sacrificing generalisability to unseen data.

**Fig. 5:**
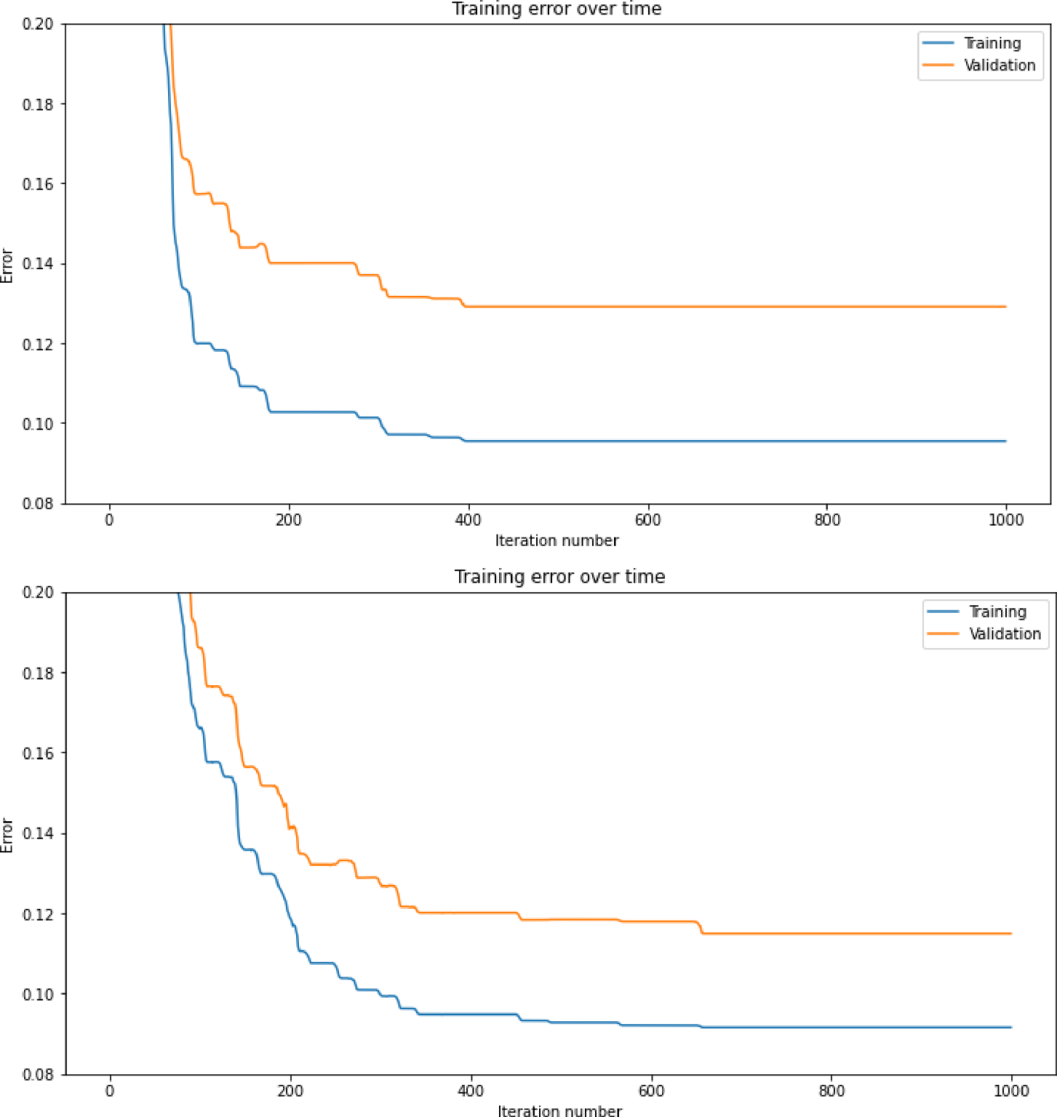
Plotting whether overfitting occurs during two consecutive runs of the evolution process for forecasting CGM data.

Relying solely on the testing dataset for early stopping can inadvertently lead to overfitting, as the model adjusts to perform optimally on both the training and testing data. We introduced a validation dataset which addresses this issue. Two consecutive training runs are shown in Fig. 5, to identify whether overfitting occurred.

### Training, testing and validation datasets

With the inclusion of a validation dataset, the data was divided into three parts: training, validation, and testing sets. The model was trained on the training set, and early stopping, parameter tuning, and model comparison are performed using the validation set. Finally, after selecting the final model architecture based on performance on the validation set, then the testing dataset was used to evaluate accuracy. By employing a validation dataset, the model does not get exposed to the testing data during training, ensuring an unbiased evaluation of its performance on unseen data. This approach helps prevent overfitting and facilitates the selection of a model that generalises well to new data. These principles aim to benefit the implementation of the genetic algorithm for the neural network to mitigate overfitting effectively.

### Genetic algorithm structure

In our implementation, each individual consists of both genotype and phenotype components. The genotype represents the actual genetic makeup of the individual, while the phenotype embodies how the individual interacts within its environment. In our specific problem context or CGM forecasting, the genotype comprises weight and bias matrices, with each matrix representing a gene in the genome. As for the phenotype, the weight and bias matrices are combined to construct a neural network within the environment. This encoding method defines the chromosome structure within the model. The fitness of the individual phenotype is defined as the accuracy of the neural network in predicting the CGM data.

For reproduction and selection processes of the genetic algorithm, we used roulette wheel selection. This method operates by establishing a cumulative distribution derived from the fitness values of individuals. Each individual’s probability of selection is determined by its fitness, with fitter individuals having a higher probability of being chosen. This selection mechanism facilitates the evolution of the population towards better-performing individuals over successive generations. For crossover, we used averaging technique, which involves taking a linear combination of the parent values. In our specific problem, the offspring weight and bias matrices will be computed as a linear combination of the corresponding matrices from the parents. This enables the weight and bias matrices to be constructed based on the parent matrices. Mutation is also applied, by adding a small random value to the weight and bias matrix for all matrices.

### Reproduction without hyper-parameters

Unlike some conventional implementations of Genetic Algorithms, this particular approach avoids the focus on hyper-parameters governing probabilities of mutation, crossover, or elitism. Instead, it simplifies the algorithm tuning process. In this approach, each set of parents generates a set of four children. These children are then pooled together with their parents, and the individual with the best fitness is selected to survive. For the offspring, all four are created through crossover using different coefficient values. However, only the last two crossover offspring undergo mutation, each with different random values. This strategy ensures algorithm convergence, as the best individual from both the offspring and parents is chosen to survive. By including both crossover and mutated individuals in the set of offspring, the need for extensive tuning of the algorithm is reduced.

### Fitness functions

The fitness function calculates the Mean Sum of Square Errors between the predicted and actual values in our time series problem as shown in Eq. (1).

1 $$MSE = \frac{{\mathop \sum \nolimits_{i}^{n} \left( {y_{i} - \hat{y}_{i} } \right)^{2} }}{n}$$

As we aim to minimise the MSE error function (Eq. 1), we need to scale our fitness values accordingly. Smaller values should yield larger scaled fitness values, while larger values should yield smaller ones. After scaling, we maximise the fitness values.

During the evolution algorithm, we fed both the training and validation data. Since we explored various window sizes, we iterated over all possible sizes, generated the data, and evaluated our algorithm accordingly. This iterative process allowed us to thoroughly test our approach across different window sizes and ensure robust performance. The algorithm operates by training on the training data and employing early stopping if the mean error for the validation data increases for three consecutive generations. Upon convergence or early stopping, the validation data is once again utilised to select the best model from the current generation.

For evaluation, we identified the model with the best validation score for each window size. Subsequently, we recreated the data based on that window size to evaluate our test MSE score. This ensured that we assessed the performance of our model across various window sizes and select the most effective one for the test evaluation.

For the testing of the genetic neural network, we execute the algorithm at each window size ranging from 3 to 10. We conduct 200 generations with a population size of 100 individuals, a maximum mutation value of 0.1, and a network architecture of [5,5,5], signifying 5 hidden nodes in 3 hidden layers. This systematic approach allows us to comprehensively evaluate the model’s performance across different window sizes and genetic configurations.

### Comparing the evolved network to a back-propogation trained neural network

For comparison of accuracy, the developed evolved neural network was compared to a standard back-propagation neural network. Ensuring a fair comparison, each neural network trained through backpropagation went through testing at various window sizes, and the best model for each run was selected based on the lowest validation dataset error. This helped to maintain consistency across the training, validation, and testing datasets for both the genetic algorithm and backpropagation methods. This approach allowed for an unbiased evaluation of the performance of each model and facilitates a meaningful comparison between the two techniques.

## Results

### Overall results for evolved neural network

**Fig. 6:**
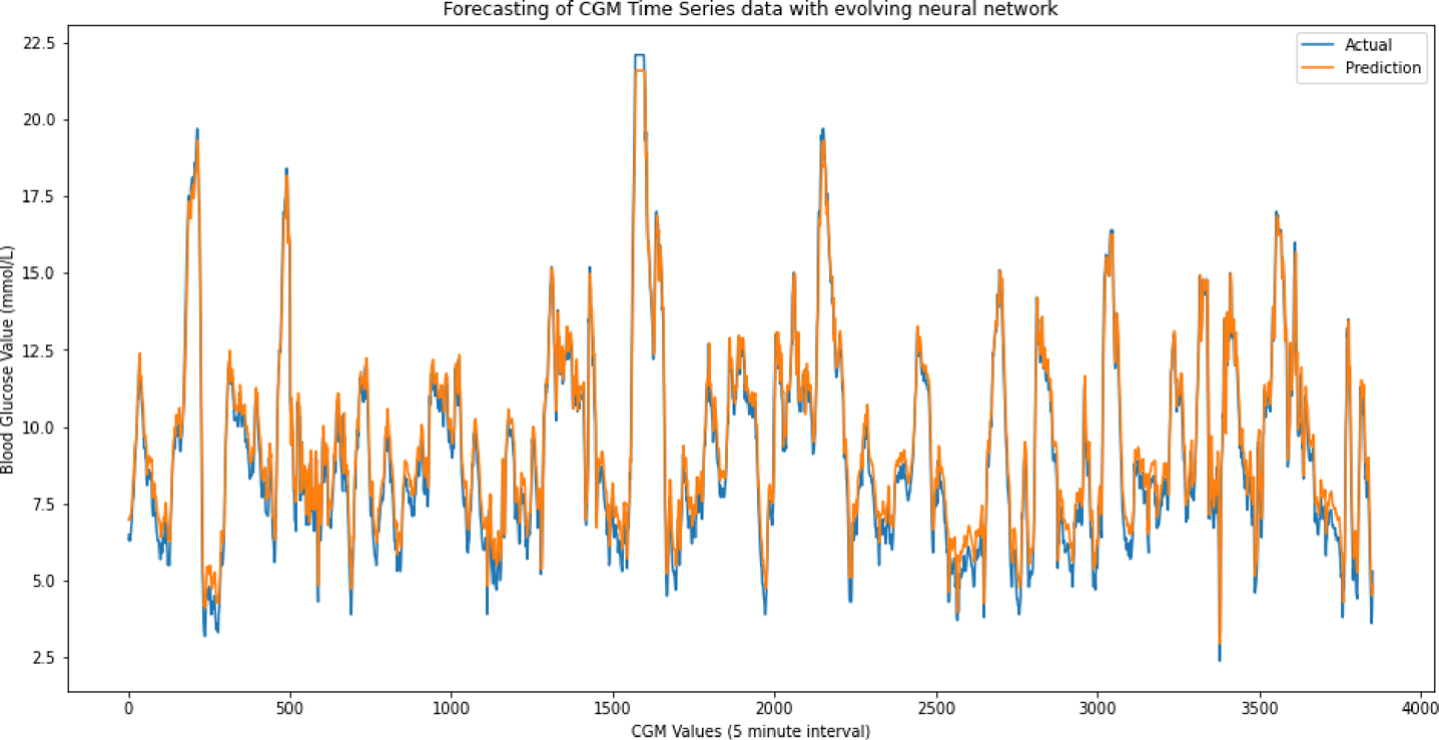
Prediction of CGM dataset using evolved neural network (neuro-evolution).

The resulting mean squared errors (MSE) for each windows size 3–10 are shown in Table 1. The visualisation in Fig. 6 provides an insight into how well the model captures the patterns and trends in the continuous glucose monitoring data.

Based on the results obtained, the model with a window size of 3 exhibits the smallest MSE for the validation dataset, making it the best choice and was selected in the final model. Upon evaluation on the test dataset, this final model yields an error of 0.105. The mean validation error remains relatively stable around 0.131, with a standard deviation of +-0.0295. Figure 6 shows the overall prediction of the entire dataset using the best model with a window size of 3.

**Table 1:** Evolved neural network accuracy of CGM forecast predictions for window sizes from 3 to 10.

Window size	Mean squared error (MSE) of CGM forecast
Window Size: 3	Validation MSE: 0.10170418080425106
Window Size: 4	Validation MSE: 0.13003596832485062
Window Size: 5	Validation MSE: 0.11094523792093151
Window Size: 6	Validation MSE: 0.1086369639253959
Window Size: 7	Validation MSE: 0.1221121629782278
Window Size: 8	Validation MSE: 0.19930775530854827
Window Size: 9	Validation MSE: 0.12489583059624539
Window Size: 10	Validation MSE: 0.15237647928675804

### Comparison to back-propogation neural network

**Fig. 7:**
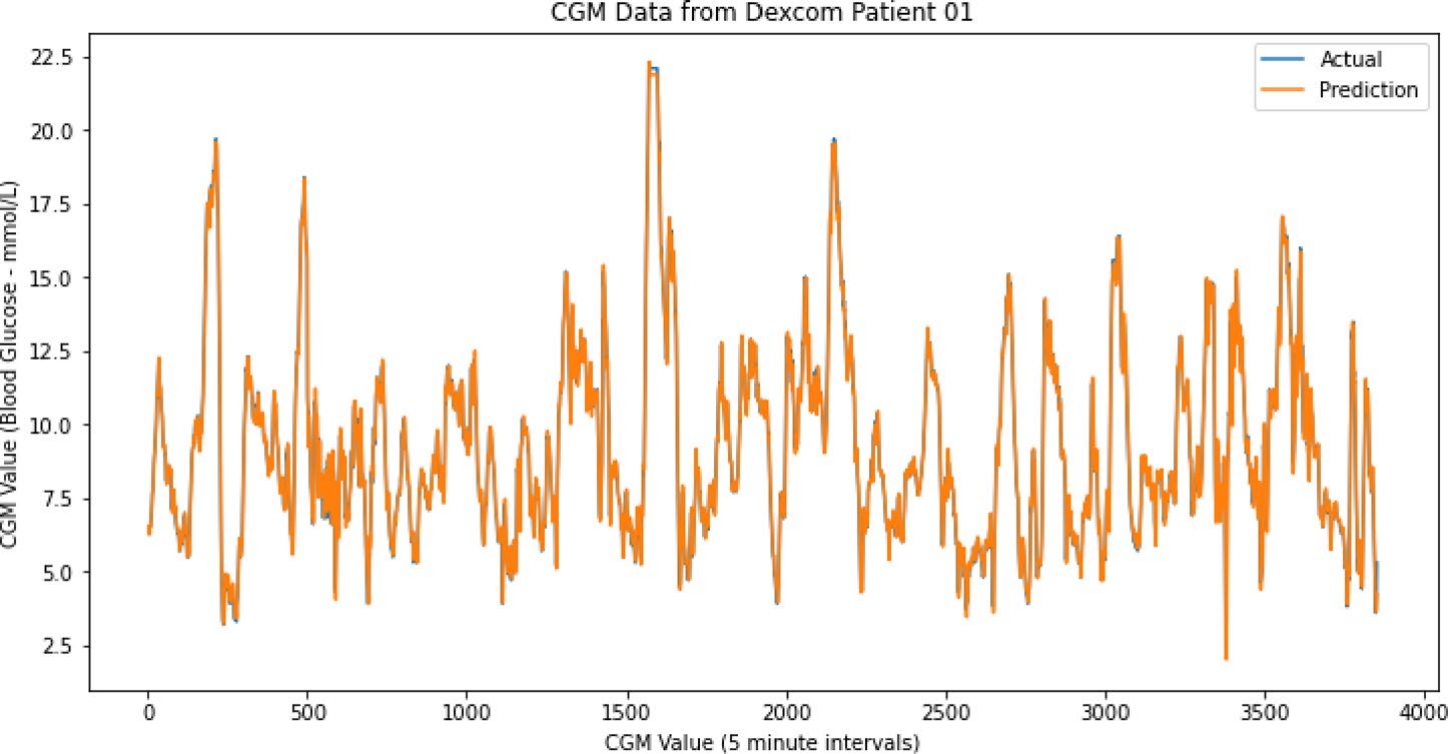
Prediction of CGM dataset using back-propogation training on the neural network.

In order to quantify improvement from using the evolutionary algorithm for neural network, the accuracy was also compared to accuracy of a standard neural network trained with back propagation. The accuracy of prediction from the back-propagation neural network is shown in Table 2. It can be seen from Table 2 that the window size in back propagation that gave lowest mean squared error was window size 5, with MSE of 0.131. Figure 7 shows the prediction for our entire time series dataset generated by the neural network trained using backpropagation.

Overall, the evolved neural network has outperformed back-propagation neural network. The evolved network achieved best MSE of 0.101 with window size 3, which outperforms the back-propagation neural network, which achieved best MSE of 0.131 with window size of 5. From the results we can see that the window size of 3 yielded the smallest validation error for evolved neural network, whereas window size of 5 yielded smallest validation error for the back-propagation neural network.

To demonstrate, clarify and quantify how much better the proposed evolving neural network approach performed (MSE 0.101) compared to the comparison back-propagation neural network approach (MSE 0.131), the absolute drop between the two approaches was MSE 0.030. This equates to relative improvement of $\:\frac{0.030}{0.131}=22.9\%$ lower MSE. If we consider RMSE to give a more interpretable result in original units $\:{RMSE}_{0.131}=0.3619\:$and $\:{RMSE}_{0.101}=0.3178\:$, which is ~ 12.2% lower RMSE (a drop of ~ 0.0441 in absolute terms). Overall, by MSE the proposed method performed ~ 23% better; in error units (RMSE) the proposed method performed ~ 12% better. However, future work can further investigate differences including between various other approaches, to ensure improvements are sustained despite the noise range and to substantiate any claim of superiority of particular approaches.

As an additional metric of performance, the back propagation neural network required a larger window size of 5, whereas the evolved neural network not only outperformed with a lower MSE, but it did so with a smaller window size of only 3. This is substantial as a window size 40% smaller further demonstrates benefits of the proposed method.

**Table 2:** Back propagation neural network accuracy of CGM forecast predictions for window sizes from 3 to 10.

Window size	Mean squared error (MSE) of CGM forecast
Window Size: 3	Validation MSE: 0.31804742177156614
Window Size: 4	Validation MSE: 10.617297686935876
Window Size: 5	Validation MSE: 0.1317070834646919
Window Size: 6	Validation MSE: 0.6893205457413865
Window Size: 7	Validation MSE: 0.1692785520201847
Window Size: 8	Validation MSE: 1.5900046103583372
Window Size: 9	Validation MSE: 0.17681231548965637
Window Size: 10	Validation MSE: 0.14451683995033807

## Discussion

Overall the accuracy of the evolved neural network outperformed the accuracy of the standard back-propagation neural network. The results demonstrated that the genetic algorithm indicates promising outcomes for prediction of continuous glucose monitoring data. Splitting the data into training, validation, and testing sets enabled the system to effectively train, compare, and evaluate our models.

Considering current limitations of this paper, the CGM datasets used were of largest size available but (1) increasing the dataset size further would be useful to include a broader demographic population, which would benefit by representing data from a wide diverse community of participants. (2) This research applied forecasting using an evolving neural network approach and compared this to back-propagation neural network forecasting, however it may benefit to include a wider range of methods including a transformer forecasting approach which has performed well, deep learning, generative AI methods, and time-series forecasting which could be completed in future to give a wider comparison between the performance of these leading methods.

Some of the possible threats to validation and limitations of the proposed model in current form, are also considered. Currently only the raw CGM values are used for training. Our previous research [Russon et al.^24^ has additionally taken account of additional external influencing factors such as meals, physical activity, or emotional states, which significantly impact blood glucose levels, by asking the participants to complete meal and exercise diaries, although accuracy of these is dependent on the participant’s own data. Future work could assess the improvement to the neuro-evolution when providing these additional activity log data, which could have potential to further enhance the forecasting capabilities.

One potential aspect of the proposed neuro-evolution method for CGM analysis is the interpretability. More interpretable models are more likely to gain acceptance by clinicians and the diabetes community. Future work can explore applying methods such as SHAP values to the proposed evolved neural network architectures, which might reduce their perceived complexity and increase their likelihood of gaining acceptance in clinical decision-making.

Fur future integration of the proposed approach into clinical decision-making, further details of the neuro-evolution process, including optimal hyperparameters, architecture search space, and evolutionary operators, could further be more thoroughly explored and documented to enable reproducibility for training with external larger datasets.

Sensor noise and missing data are further potential challenges and considerations for translating this neuro-evolutionary model into real-world clinical or personal health monitoring scenarios. Although we identified some of the missing data properties of the current dataset, further investigations especially regarding sensor noise and missing data may provide useful insights.

The current model was trained to predict one time-step ahead which gives a 5-minute prediction horizon. Future work can apply the proposed methods to provide predictions of different windows sizes, for example a prediction horizon of 30 min, 60 min–2 h. It is commonly considered that 4 h is around the longest CGM prediction horizon that can be regularly attempted.

Given the computational demands of neuro-evolution, the practical use for this approach for real-time or large-scale clinical applications could be by using a trained model on unseen data to forecast the glucose values. Future research could be conducted to compare the computational demands between the proposed method and other conventional approaches such as back propagation.

Additional future research could further this investigation by assessing how the robustness of the proposed method would be affected when applied to unseen or external datasets.

Further future work can involve comparing this proposed neuro-evolution approach with other advanced time series forecasting models and modern baselines, such as LSTM or GRU networks which are performing well in other areas. To boost reproducibility we have shared the data and code for future researcher experimentation. Additional future work could develop cross-patient validation or feature normalization to further refine evaluations.

## Conclusion

In the domain of computational intelligence, the application of genetic algorithms to neural networks constitutes the sub-field of neuro-evolution applied in this project. Neuro-evolution encompasses various approaches, wherein not only the weights but also the architecture and hyper-parameters of the neural network can be evolved. This neuro-evolution paradigm is particularly advantageous for reinforcement-type problems where the fitness function is non-differentiable, rendering backpropagation inapplicable, or when dealing with small-scale neural networks, as the ANN topology used in this research. However, it’s important to note that Neuro-evolution would not be the optimal approach for training Deep Neural Networks (DNNs) due to their vast number of parameters. Evaluating each weight matrix through crossover and mutation has been previously shown to become computationally intensive, scaling rapidly with the number of weights, as it takes O(n*m) to evaluate each weight matrix.

Overall, neuro-evolution remains a pertinent area of interest, particularly for evolving both the architecture (topology) and hyper-parameters (and weights) of neural networks. This approach is also known as Auto-Machine Learning, which aims to automate the process of algorithm tuning, leveraging genetic algorithms to discover the optimal set of hyper-parameters and architecture for a given neural network. This eliminates the need for manual tuning by data scientists and streamlines the model development process.

Future research could investigate larger CGM datasets, consisting of a greater number of individuals. Also, it may be worth evaluating these methods on CGM data with differing time recording intervals, such as 1-, 5- and 15-minute intervals to see if the recording interval affects prediction accuracy, as the CGM has previously been shows to affect metrics such as the number of hypoglycaemic episodes [Russon et al.^3,4^. Further work could investigate further why these window sizes were optimal for the particular training methods.

## Data Availability

Python code developed during the current study and an example data file for analyis are available from the corresponding author on reasonable request. This consists of the anonymous CGM data file containing column 1: sequential index from 0 to 3852, column 2: timestamp at 5 min intervals, column 3: CGM value as mmol/L to 1 decimal place. The Python Jupyter Notebook file contains the code used to train the model and produce the graphs included in this article.
